# Localisation and substrate specificities of transglycanases in charophyte algae relate to development and morphology

**DOI:** 10.1242/jcs.203208

**Published:** 2018-01-15

**Authors:** Klaus Herburger, Louise M. Ryan, Zoë A. Popper, Andreas Holzinger

**Affiliations:** 1 Department of Botany, Functional Plant Biology, University of Innsbruck, Sternwartestraße 16, 6020 Innsbruck, Austria; 2 Botany and Plant Science and Ryan Institute for Environmental, Marine, and Energy Research, School of Natural Sciences, National University of Ireland, Galway, University Road, H91 TK33 Galway, Ireland

**Keywords:** Charophyte green algae, Mixed-linkage β-glucan, Transglycosylation, Xyloglucan transglucosylase hydrolase, Xylan, Xyloglucan

## Abstract

Cell wall-modifying enzymes have been previously investigated in charophyte green algae (CGA) in cultures of uniform age, giving limited insight into their roles. Therefore, we investigated the *in situ* localisation and specificity of enzymes acting on hemicelluloses in CGA genera of different morphologies and developmental stages. *In vivo* transglycosylation between xyloglucan and an endogenous donor in filamentous *Klebsormidium* and *Zygnema* was observed in longitudinal cell walls of young (1 month) but not old cells (1 year), suggesting that it has a role in cell growth. By contrast, in parenchymatous *Chara*, transglycanase action occurred in all cell planes. In *Klebsormidium* and *Zygnema*, the location of enzyme action mainly occurred in regions where xyloglucans and mannans, and to a lesser extent mixed-linkage β-glucan (MLG), were present, indicating predominantly xyloglucan:xyloglucan endotransglucosylase (XET) activity. Novel transglycosylation activities between xyloglucan and xylan, and xyloglucan and galactomannan were identified *in vitro* in both genera. Our results show that several cell wall-modifying enzymes are present in CGA, and that differences in morphology and cell age are related to enzyme localisation and specificity. This indicates an evolutionary significance of cell wall modifications, as similar changes are known in their immediate descendants, the land plants.

This article has an associated First Person interview with the first author of the paper.

## Abstract (precis)

**Summary:** The distribution of major hemicelluloses and cell wall-modifying enzymes in three charophyte green algae (CGA), representing the ancestors of land plants, relate to algal morphology and developmental stage.

## INTRODUCTION

A shared feature of plants and most green algae is that their cells are surrounded by cell walls, which are a diverse composite of complex polysaccharides and crucial for plant function and survival (Popper et al., 2011). In particular, walls of late diverged charophyte green algae (CGA, e.g. Zygnematophyceae, Charophyceae) and land plants exhibit chemical similarities, while more ancient CGA (e.g. Klebsormidiophyceae) lack some of the components found in their descendants (Table S1). This supports the hypothesis that the entire land plant lineage evolved from a single group within the CGA, namely the Zygnematophyceae (Wickett et al., 2014), which were able to colonize terrestrial habitats about 460 million years ago (Becker and Marin, 2009). Consequently, it has been proposed that land plants inherited the major cell wall components from their algal ancestors (Domozych et al., 2012) with a cell wall considered a prerequisite for terrestrial survival (Harholt et al., 2016). It has been shown recently that flexible cell walls mediated by desiccation-induced callose deposition in *Klebsormidium* (Herburger and Holzinger, 2015) or the specific occurrence of pectic substances in the macroalgae *Ulva compressa* (Holzinger et al., 2015) coincide with elevated desiccation tolerance in aero-terrestrial or intertidal habitats, respectively. This suggests that modulating the cell wall architecture and composition in response to abiotic stress was crucial for the survival of algal colonizers of terrestrial habitats. Although the cell walls of various CGA have been explored over the past decades, there are many remaining questions regarding the localisation and metabolism of specific wall components.

Polysaccharides of plant cell walls are synthesized by glycosyltransferases (GTs) within Golgi bodies (hemicelluloses and pectins) or at the plasma membrane (cellulose and callose) and are secreted into the cell wall (Scheller and Ulvskov, 2010; Harholt et al., 2010). In plant cell walls, specific enzymes modify the hemicelluloses, for example by hydrolysis or transglycosylation (Franková and Fry, 2013). Hemicelluloses are a group of polysaccharides that interact, typically through hydrogen bonds, with cellulose microfibrils (Carpita and Gibeaut, 1993; Park and Cosgrove, 2012). While hydrolases cleave glycosidic bonds in the backbone of cell wall polysaccharides (e.g. the β-1→4-bond between d-glucopyranose residues in xyloglucan), transglycosylases cut a polysaccharide chain (donor) and reattach it to an acceptor substrate (Rose et al., 2002). The latter can be either an endogenous cell wall polysaccharide or an exogenous oligosaccharide (Fry, 1997). Xyloglucan is one of the most abundant hemicelluloses in the primary cell walls of non-commelinid flowering plants (Fry, 2011). Processing by xyloglucan endotransglucosylase hydrolase (XTH; EC 2.4.1.207) aids the incorporation of newly synthesized xyloglucan into the cell wall (Thompson et al., 1997), loosening of cell walls during expansive cell growth (Fry et al., 1992; Van Sandt et al., 2007), shrinkage of tension wood fibres in trees in response to gravitropism (Nishikubo et al., 2007), and fruit growth and ripening (Han et al., 2015). Other donor substrates for transglycosylases are mannans, mixed-linkage (1→3,1→4)-β-d-glucan (MLG), cellulose and, to a lesser extent, xylans (Schröder et al., 2004; Fry et al., 2008a; Simmons et al., 2015; Shinohara, et al., 2017). Transglycosylation activity between xyloglucan and either xyloglucan (xyloglucan:xyloglucan endotransglucosylase activity; XET) or MLG (MLG:xyloglucan endotransglucosylase activity; MXE) has also been demonstrated in extracts of some charophytes *in vitro* (Fry et al., 2008a). Furthermore, blotting algal thalli onto paper coated with sulphorhodamine-labelled xyloglucan oligosaccharides (XyGO-SRs) (tissue prints) suggested that there was transglycosylase activity in vitro in growth zones of the macroalgae *Chara* (Charophyta) and *Ulva* (Chlorophyta) (Van Sandt et al., 2007a). While the tissue-printing technique provides a good spatial estimation of transglycosylase activities at the tissue level (e.g. Olsen et al., 2016), it is less precise than *in vivo* techniques that are able to resolve enzyme action at the cellular level (Vissenberg et al., 2000). For green algae, the resolution of transglycosylase action at the cellular level is still missing. This has resulted in a considerable knowledge gap, particularly for filamentous and unicellular green algae that are too small for the tissue-printing technique to be applied. Knowledge of the precise spatiotemporal localisation of wall-modifying enzymes would provide valuable new insights into the mechanisms of cell growth in simple multicellular plants.

The present study focuses on three members of the CGA, *Klebsormidium*, *Zygnema* and *Chara*. The latter forms morphologically complex thalli and grows in the water body of lakes and ponds, while filamentous *Klebsormidium* and *Zygnema* occur worldwide in limnic and aero-terrestrial habitats and fulfil numerous important ecological functions as components of biological soil crusts (Elbert et al., 2012). With increasing age, cell walls of *Zygnema* and *Klebsormidium* undergo dramatic changes, such as an increase in diameter and the formation of additional layers (Mikhailyuk et al., 2014; Herburger et al., 2015; Pichrtová et al., 2016a). However, information is scarce regarding whether these morphological changes also involve changes in the chemical composition of the cell wall or the activity and specificity of cell wall-modifying enzymes. To date, algal cell or filament age as a factor influencing the architecture and composition of the cell wall, has received little attention. This is surprising since cell wall composition and the hemicelluloses (e.g. xyloglucan, mannans) incorporated into the wall are known to be altered in response to cell age (Métraux, 1982; Morrison et al., 1993). We investigated the donor substrate specificity and localisation of transglycanases *in vitro* and *in vivo*. This is the first study showing both the location of the transglycosylase action *in vivo* and at the cellular level in charophyte algae. Long-term cultivation experiments (up to 1 year) allowed us to compare enzyme activity/action in algae of different culture age and cells of different developmental stages. Based on observations of algal populations in various hydro- and aero-terrestrial habitats (e.g. Karsten et al., 2010; Pichrtová et al., 2014) and cultured algae (e.g. Herburger et al., 2015), we hypothesized that cell age changes: (1) the architecture of the cell wall (i.e. distribution of hemicelluloses), and (2) the activity and specificity of enzymes acting on cell wall polysaccharides. Possible biological functions of different hemicelluloses as well as implications for the high ecophysiological and evolutionary success of these algae are discussed.

## RESULTS

### Age-dependent cell wall thickening correlates with higher proportion of pectins or hemicelluloses

**Fig. 1.**
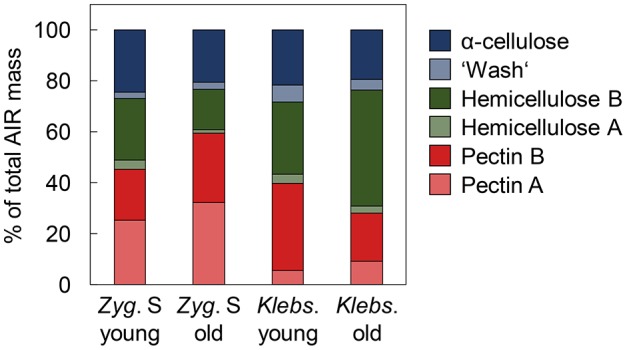
**Fractionation of cell wall components from young and old *Zygnema* S and *Klebsormidium crenulatum* filaments into six classes.** Classes are shown as percentages of total alcohol-insoluble residue (AIR). *n*=3 (s.d. <5%). *Zyg*. S, *Zygnema* S; *Klebs*., *Klebsormidium.* Young filaments are 1 month old; old filaments are 12 months old.

To test whether increasing cell age (1 month compared to 1 year) changes the cell wall composition of *Klebsormidium* and *Zygnema* S, the alcohol-insoluble residue (AIR) of algal filaments was fractionated (Fig. 1) and analysed. *Zygnema* S filaments possessed a larger pectin fraction when compared with *Klebsormidium*, with highest amounts being found in old filaments. In contrast, increasing cell age increased the total hemicellulose content of *Klebsormidium*. This suggests that age-dependent cell wall thickening in *Zygnema* is characterized by an increase in the pectin content, while in *Klebsormidium* cell walls are thickened by deposition of hemicelluloses.

### *Zygnema* S transglycanases accept a wider range of donor substrates than *Chara* and *Klebsormidium in vitro*

**Fig. 2.**
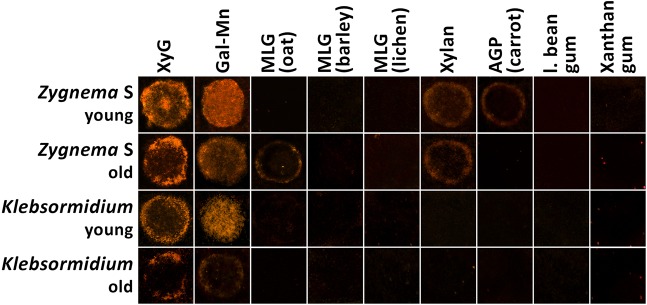
**Dot-blot assay for transglycosylase activity of enzyme extracts of young and old *Zygnema* S and *Klebsormidium crenulatum* filaments.** Test papers were coated with 1% (w/v) solutions of different cell wall polysaccharides and ∼5 mM XyGO-SR. Enzyme extracts (in 5 µl aliquots), were loaded on the test papers and incubated for 2 h before washing with an ethanol:formic acid:water (1:1:1, v/v/v) mixture for 2 h, rinsing twice with distilled water and drying overnight. XyGO-SR was visualized at 365 nm. Young filaments are 1 month old; old filaments are 12 months old.

To estimate whether different hemicellulose contents in *Klebsormidium* and *Zygnema* S and in young and old filaments coincide with different substrate specificities of hemicellulose-modifying enzymes, a dot-blot assay testing the transglycosylase activities of extracted algal proteins was carried out. Extracts from freshly isolated *Chara* thalli were also analysed, but only confirmed previous results showing transgylcosylase activity between xyloglucan:xyloglucan and xyloglucan:MLG (e.g. Fry et al., 2008a). *Zygnema* S extracts exhibited transglycosylase activity towards all major hemicelluloses tested [xyloglucan, galactomannan, MLG (old extracts), xylan] and arabinogalactan proteins (AGPs; young extracts) (Fig. 2, Table 1). In contrast, the activity of extracts from *Chara* and *Klebsormidium* was restricted to xyloglucans and galactomannan only. *Klebsormidium* extracts showed the lowest detection signals (Table 1).

**Table 1.**
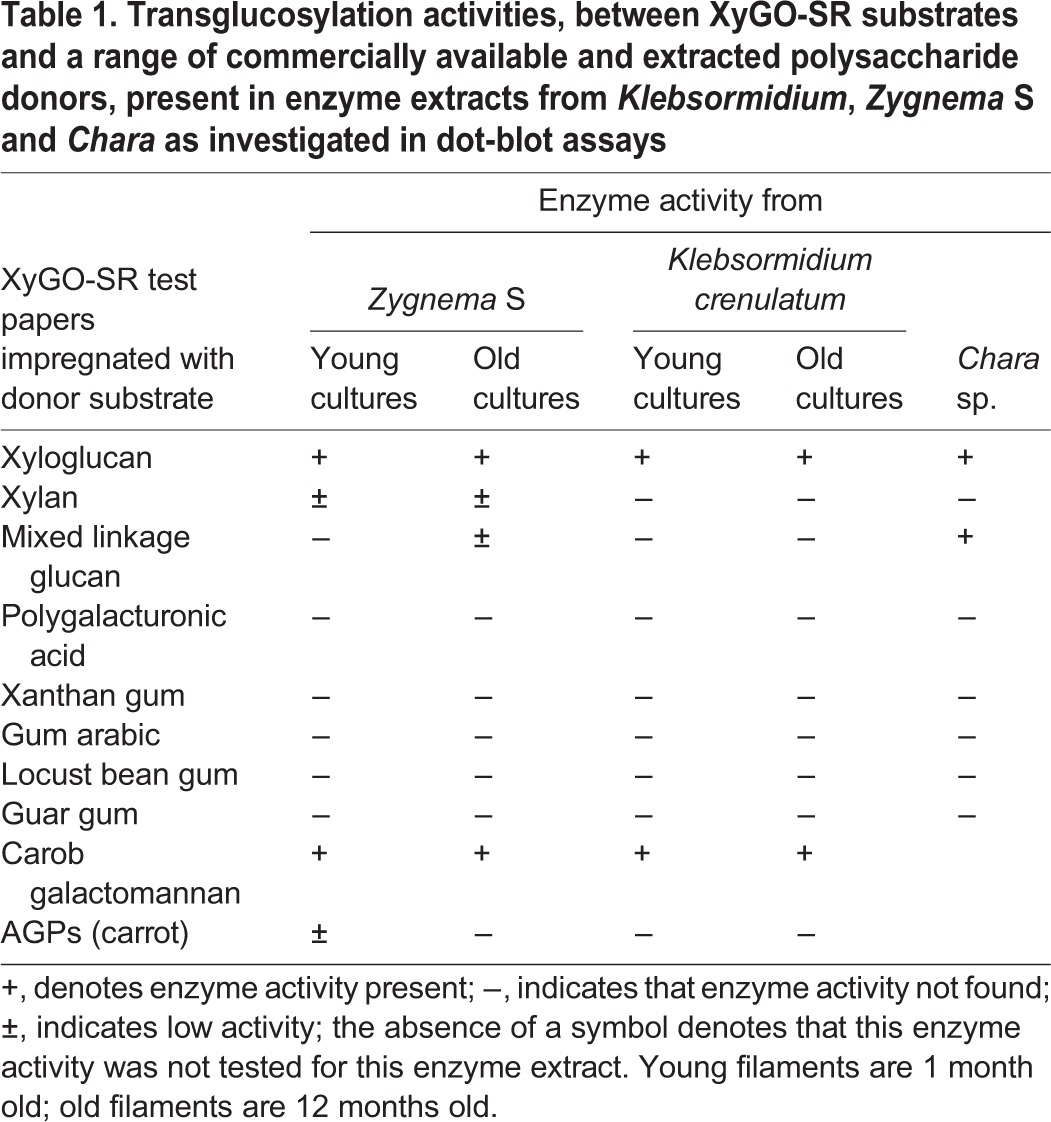
**Transglucosylation activities, between XyGO-SR substrates and a range of commercially available and extracted polysaccharide donors, present in enzyme extracts from *Klebsormidium*, *Zygnema* S and *Chara* as investigated in dot-blot assays**

### Transglycosylation between xyloglucan and endogenous donor substrates occurs in charophyte cell walls *in vivo*

**Fig. 3.**
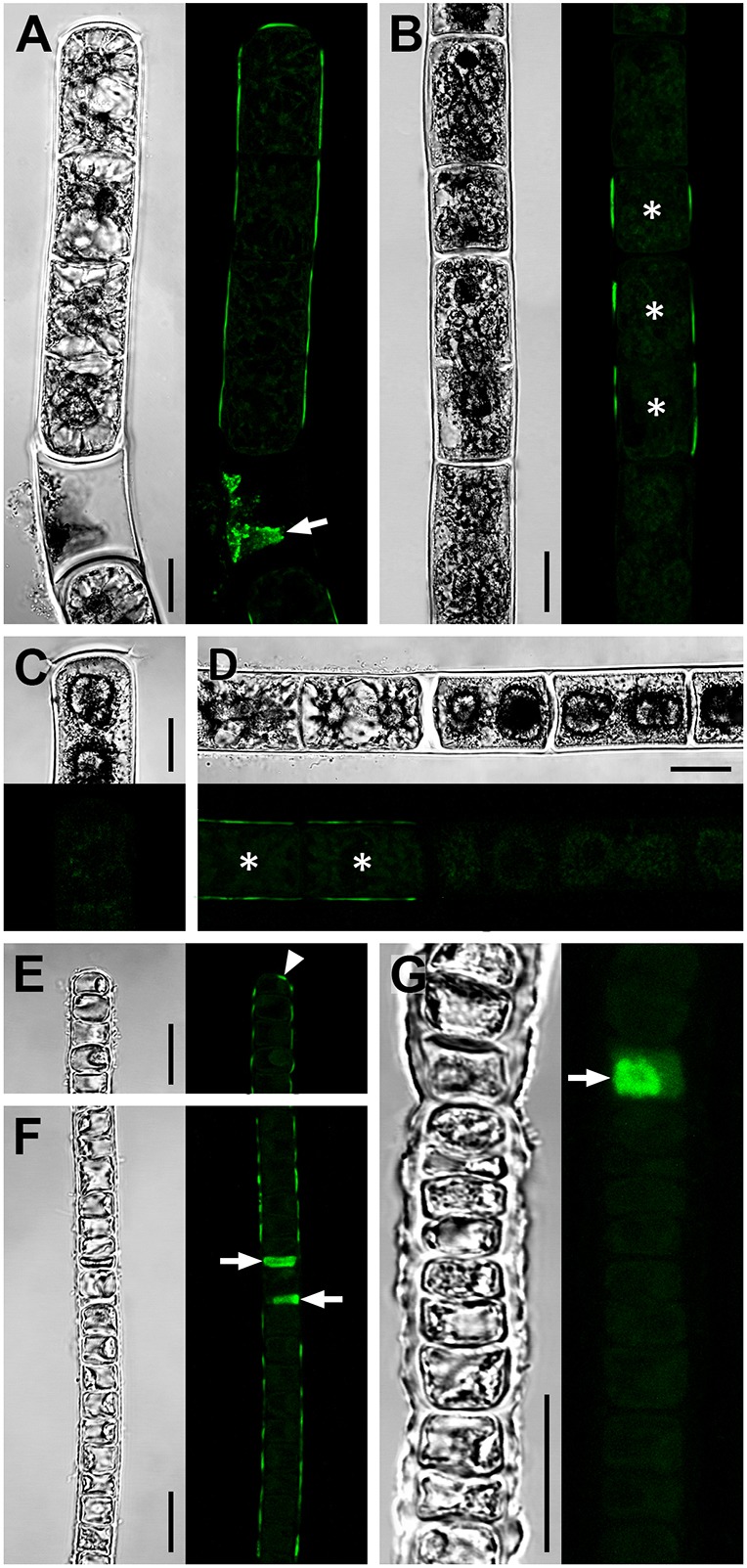
**Transglycosylase action in young and old *Zygnema* S and *Klebsormidium crenulatum* filaments.** Confocal micrographs showing integration of the fluorescent acceptor substrate XyGO-SR in young (A,E,F) and old (B–D,G) filaments of *Zygnema* S (A–D) and *Klebsormidium crenulatum* (E–G) indicative of transglycosylase action. After XyGO-SR incorporation, filaments were incubated in DMF to remove chlorophyll autofluorescence. Cells that are dead prior to XyGO-SR incubation were seen to contain fluorescent cytoplasmic residue (A,F,G; arrows). Corresponding bright-field images are also shown. (A) Filament with fluorescence in outer cell walls and a terminal cross cell wall. (B) Filament showing fluorescence in longitudinal cell walls of short cells (asterisks), but not in longer cells. (C) Terminal cell lacking fluorescence. (D) Filament with fluorescence in outer walls of two vegetative cells (asterisks) but not in adjacent pre-akinetes. (E,F) Filaments with fluorescence in outer cell walls including a terminal cross cell wall (arrowhead). (G) Filament lacking fluorescence in cell walls. Young filaments are 1 month old; old filaments are 12 months old. Scale bars: 10 µm.

**Fig. 4.**
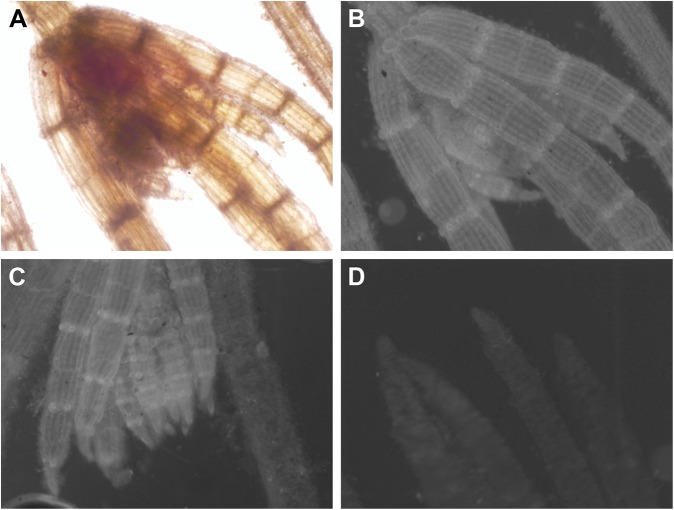
**Fluorescence microscopy images showing integration of the fluorescent acceptor substrate XyGO-SR into *Chara* sp. cell walls.** After incubation in XyGO-SRs, *Chara* was washed in culture medium and viewed using the DAPI channel of an epifluorescence microscope at ×40 magnification. (A) Bright-field image. (B,C) Incorporation of XyGO-SRs in all cell walls. The walls of the stipulodes, and the cells towards the tip of the main axis and branchlets appeared to have incorporated the most XyGO-SRs and fluoresced the most strongly. (D) Control in which *Chara* sp. was incubated with non-fluorescent XyGOs.

Since transglycosylase activity (*in vitro*) was found in all charophytes analysed, algae were exposed to fluorescent XyGO-SRs to test for transglycosylase action (*in vivo*). Both, young *Zygnema* S and *Klebsormidium* filaments incorporated XyGO-SR fluorescence into their outer cell walls, including terminal cross cell walls, but not in the cell corners between individual cells or in the inner cross cell walls (Fig. 3A,E,F). This highly specific occurrence of enzyme action was also found in younger, growing cells and occasionally occurred in old *Zygnema* S filaments, but was not seen in the majority of cells (i.e. thick-walled pre-akinetes) within the same filaments (Fig. 3B). Old *Klebsormidium* filaments with thick cell walls were predominantly devoid of fluorescence (Fig. 3E) with the exception that some filaments contained dead cells that exhibited strong auto-fluorescence derived from cytoplasmic residue. The autofluorescence associated with dead cells is distinct from XyGO-SR fluorescence and was also observed in dead cells of control filaments (data not shown). In contrast to *Zygnema* S and *Klebsormidium*, parenchymatous *Chara* sp. incorporated XyGO-SRs into all cell planes with a maximum in younger cells towards the apex of the main axis and the branchlets, and in the walls of the stipulodes (Fig. 4).

### Highly complex cell wall composition

**Fig. 5.**
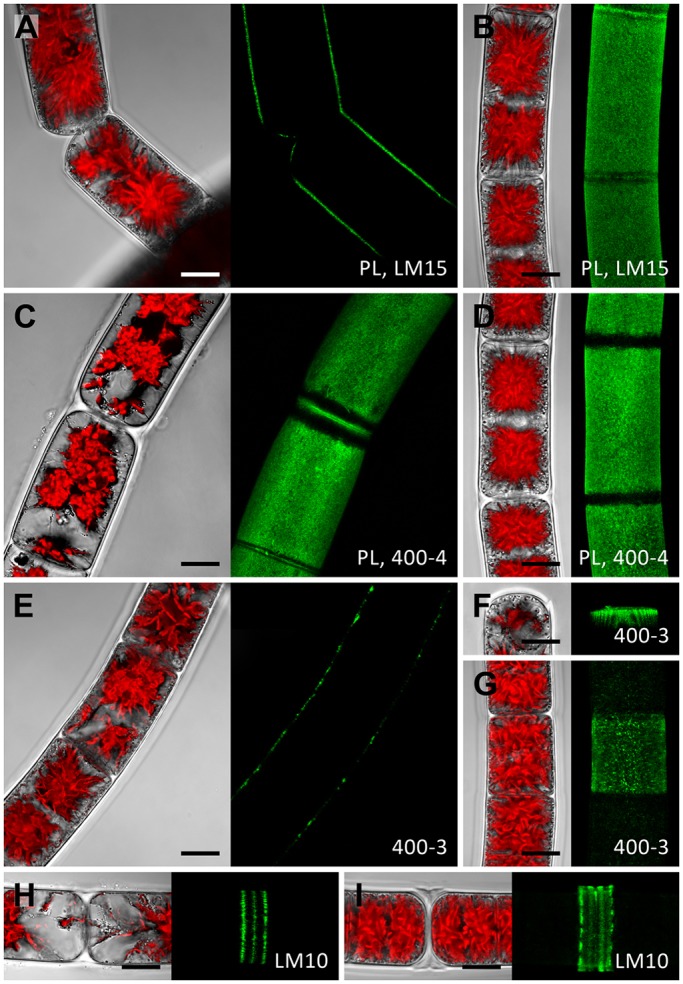
**Whole-cell labelling of *Zygnema* S.** Young (A,C,E,F,H) and old (B,D,G,I) filaments labelled with the monoclonal antibodies LM15, 400-4, 400-3 or LM10 (green). In the confocal micrographs in A and E, one optical section is shown, confocal micrographs in B–D and F–I show *z*-projections of ∼50 optical sections. The corresponding bright-field images include red chloroplast autofluorescence. (A) Detaching cells with staining in exposed cell walls. (B) Staining in outer and cross cell walls but not in ribbon-like zones close to cross cell walls. (C) Similar pattern to that shown in B. (D) Staining in outer cell walls. (E) Filament with patchy labelling in outer cell walls. (F) Circular staining underneath expanded terminal cross wall. (G) Central cell showing patchy straining, which is weak in adjacent cells. (H) H-shaped cell wall structure with staining in three distinct rings. (I) Prominent H-shaped cell wall structure with strong staining. Young filaments are 1 month old; old filaments are 12 months old. Scale bars: 10 µm.

**Fig. 6.**
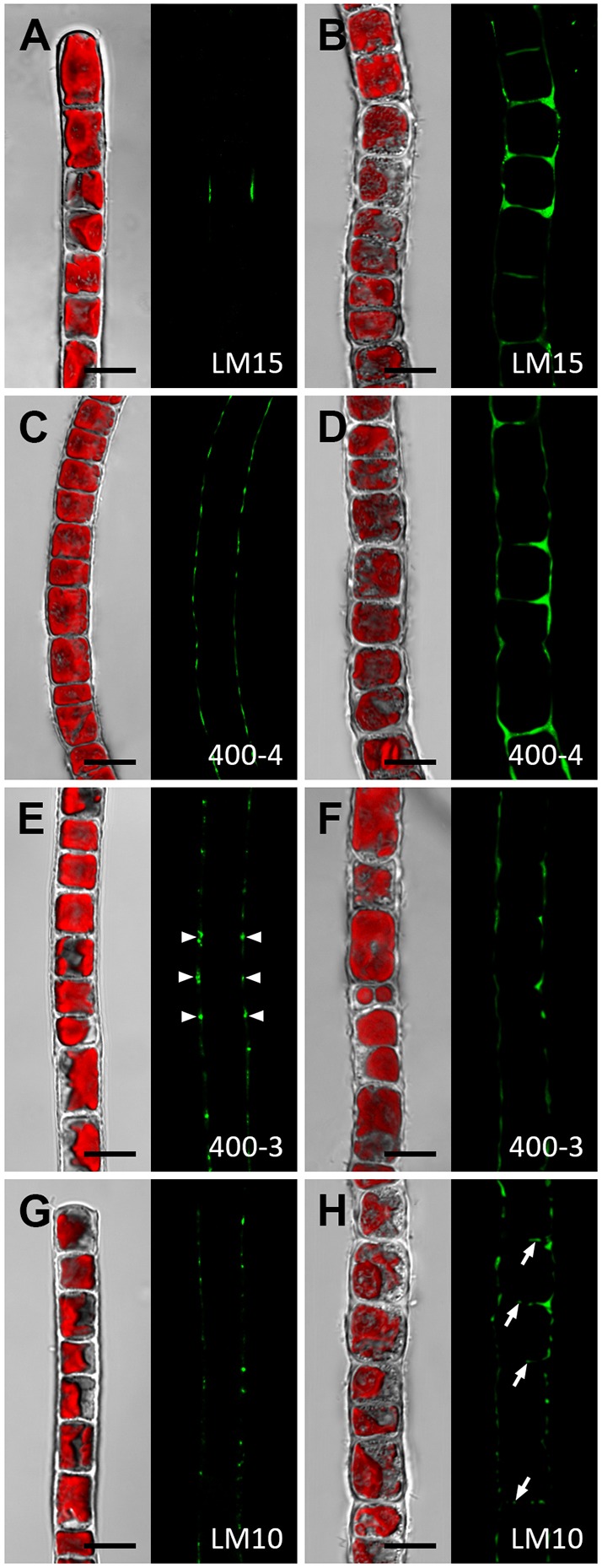
**Whole-cell labelling of *Klebsormidium crenulatum*.** Young (A,C,E,G) and old (B,D,G,H) filaments labelled with the monoclonal antibodies LM15, 400-4, 400-3 or LM10 (green) and visualised with a confocal microscope. The corresponding bright-field images include red chloroplast autofluorescence. (A) Filament with weak staining in restricted areas. (B) Intense labelling in thickened cell corners between individual cells and some staining in cross cell walls. (C) Staining in outer cell walls. (D) Intense staining in cell corners and thickened cross cell walls. (E,F) Staining in cell corners (arrowheads) and occasionally in longitudinal cell walls of longer cells. (G) Punctuate staining pattern in outer cell walls. (H) Similar appearance to cells shown in G; additionally, cross cell walls show staining (arrows). Young filaments are 1 month old; old filaments are 12 months old. Scale bars: 10 µm.

Colocalisation of XyGO-SRs and transglycosylase action does not provide information regarding the presence of potential endogenous donor substrates. Therefore, a set of cell wall polymer-specific monoclonal antibodies (mAbs) was used to generate a spatial map for hemicellulose distribution in the cell walls of *Zygnema* S (Fig. 5) and *Klebsormidium* (Fig. 6). We labelled whole cells (Figs 5 and 6; Fig. S2A,B) and sections of high-pressure frozen filaments, the latter exposing cross cell walls directly to the mAbs (Fig. S2C–F). Results are summarized in Fig. S3. The outer and cross cell walls of young and old *Zygnema* S filaments (Fig. 5A–D, in Fig. 5A one optical section is shown, whereas in Fig. 5B–D *z*-projections of ∼50 optical sections are shown; Fig. S2C,D) labelled with LM15 (which recognises epitopes present in xyloglucan) and mAb 400-4 [which recognises epitopes present in (1→4)-β-mannans] colocalised with areas of XyGO-SR incorporation. Old filaments lacked mannan epitopes in cross walls (Fig. 5D). In contrast, MLG epitopes (mAb 400-3), showed a punctate labelling pattern in outer cell walls of young filaments (Fig. 5E, one optical section shown). Occasionally, MLG (mAb 400-3) was labelled as a band close to the expanded terminal cross cell walls in young *Zygnema* S filaments (Fig. 5F, *z*-stack) whereas old *Zygnema* S filaments showed stronger MLG labelling in some cells (Fig. 5G, *z*-stack). Xylan epitopes (mAb LM10) were restricted to H-shaped cell wall structures in both young and old *Zygnema* S filaments (Fig. 5H,I, *z*-stacks).

In contrast to *Zygnema* S, xyloglucan (mAb LM15) and mannan epitopes (mAb 400-4) were scarce in young *Klebsormidium* filaments (Fig. 6A,C; in Fig. 6 single optical sections are shown) with more intense labelling observed in the thickened cell walls of old filaments (Fig. 6B,D). MLG epitopes (mAb 400-3) were restricted to the cell corners between individual *Klebsormidium* cells (Fig. 6E,F) and xylan epitopes (mAb LM10) showed a punctuate distribution in outer cell walls with occasional labelling of the cross cell walls of old *Klebsormidium* filaments (Fig. 6H).

Pectate lyase (PL) treatment increased the strength of the antibody signal (Fig. S2A,B), but neither altered labelling patterns nor facilitated detachment of cells [i.e. single cells or small filaments (2–5 cells) were not enriched] in either *Zygnema* S or *Klebsormidium* (data not shown).

### Transglycosylase activity changes with culture age

**Fig. 7.**
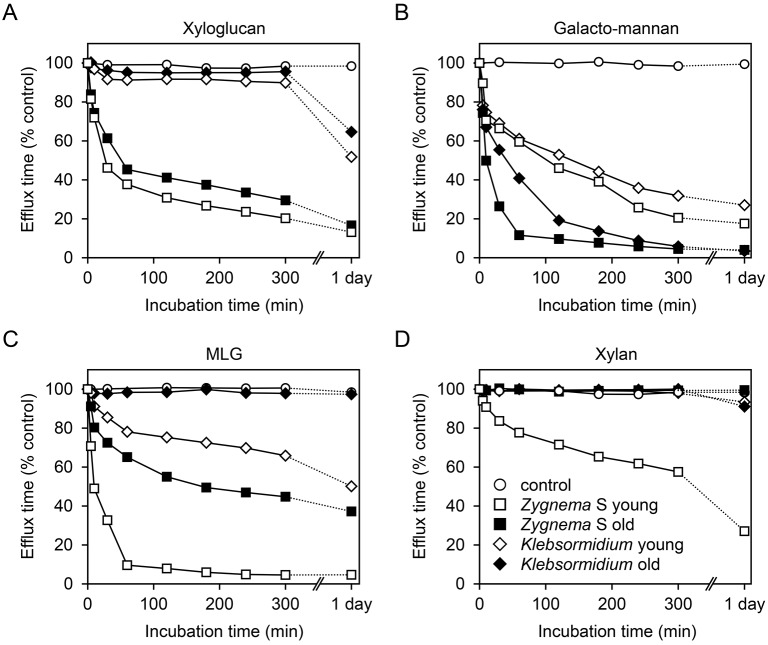
**Viscometric assay estimating the hydrolysis of four cell wall polysaccharides by enzyme extracts of young and old *Zygnema* S and *Klebsormidium crenulatum* filaments.** Reaction mixtures contained 1% (w/v) polysaccharide, 300 mM Na succinate (pH 5.5) and 10% (v/v) dialysed algal enzyme extract. (A) Xyloglucan, (B) galactomannan, (C) mixed-linkage (1→3,1→4)-β-glucan (MLG) from oat, (D) xylan. *n*=3 (s.d. <5%). Young filaments are 1 month old; old filaments are 12 months old.

To test whether transglycosylase activities of extracts prepared from young and old algal filaments was accompanied by hydrolytic activity, the loss of viscosity of four different polysaccharide solutions was investigated (Fig. 7). Hydrolysis of xyloglucan was strongest after adding extracts prepared from young or old *Zygnema* S filaments, where the efflux time decreased to ∼13–16% after 1 day (Fig. 7A). In contrast, hydrolysis of galactomannan was greatest following treatment with extracts prepared from old *Zygnema* S and *Klebsormidium* filaments, decreasing efflux time to ∼5% after 5 h (Fig. 7B). Extracts from young *Zygnema* S filaments showed the highest hydrolytic activity towards MLG, reducing efflux time to <10% within 1 h (Fig. 7C). Extracts from young *Klebsormidium* filaments showed lower hydrolytic activity, which was absent in old *Klebsormidium* (Fig. 7C). Hydrolysis of xylan was only observed after adding young *Zygnema* S extracts and the efflux time decreased to ∼25% after 1 day (Fig. 7D).

## DISCUSSION

The present study provides new insights into algal cell wall metabolism by showing that transglycosylase actions are abundant in young filaments of both *Klebsormidium* and *Zygnema* S and restricted to longitudinal cell walls, where cell expansion occurs. In contrast, enzymatic actions decrease with increasing filament age. *In vitro* studies and immunolocalisation of the major hemicelluloses (xyloglucan, mannans, MLG and xylan) suggested that xyloglucan and mannans (*Zygnema* S) or xyloglucan (*Klebsormidium*) are the most likely donor substrates. These results show for the first time, that the hemicellulose network of early (*Klebsormidium*) and late (*Zygnema*) diverged CGA undergoes strong spatiotemporal changes and might be involved in survival strategies such as pre-akinete formation, regularly observed in *Zygnema*.

### Cell wall composition changes with cell age

Land plant cell walls are remodelled in response to ageing and stresses (e.g. mechanical or chemical stresses). In contrast, the effect of cell age on the cell wall composition of CGA members had not been investigated, even though, for example, increasing cell age and environmental stress triggers the formation of resistant pre-akinetes in *Zygnema*, which are crucial for survival and involve changes of the cell wall morphology such as massive thickening (e.g. McLean and Pessoney, 1971; Herburger et al., 2015). As found in the present study, age-dependent cell wall thickening in *Zygnema* S predominantly consists of an increase in the pectin content, while in *Klebsormidium* cell walls are thickened by deposition of hemicelluloses.

However, in both genera, immunostaining of whole cells and sections of high-pressure frozen filaments with a set of cell wall polymer-specific monoclonal antibodies (mAbs) revealed an increased abundance of hemicellulose epitopes in thickened cell wall parts. In the case of *Zygnema* S, this was particularly true for MLG and xylan, while xyloglucan and mannan were detectable in both old and young filaments and had levels that were independent of cell size. This suggests that the latter two hemicelluloses are important cell wall components in the species investigated. Both xyloglucan and mannans are associated with homogalacturonan, since the labelling signal strongly increased upon unmasking with PL. This underpins the close relationship between Zygnematophyceae and land plants, where close physical proximity between xyloglucan and pectins, indicating stable interactions, were confirmed by 2D and 3D solid-state nuclear magnetic resonance (ssNMR) (Dick-Pérez et al., 2011). A considerable amount of xyloglucan might be covalently linked to pectins as shown for a range of angiosperm cell suspension cultures (Thompson and Fry, 2000; Popper and Fry, 2008). The restriction of xylan epitopes to thickened cell wall areas in both *Zygnema* S and *Klebsormidium* (H-shaped structures between individual cells) resembles findings in land plants (e.g. *Nicotiana tabacum*), where xylans can be abundant in thickened primary cell walls of collenchyma and epidermis cells (Hervé et al., 2009). As shown recently, land plant xylan interacts tightly with cellulose (Simmons et al., 2016) and its absence impairs cell wall strength and the vessel development. A role of xylan in strengthening algal cell walls is suggested by an increased binding of LM10 to old and thickened *Klebsormidium* cell walls, while young filaments that are characterised by highly flexible cell walls (Herburger and Holzinger, 2015) showed less binding. Detection of xylans in the early diverged CGA *Klebsormidium* reflects the occurrences of xylan metabolism-related genes in the recently published *Klebsormidium flaccidum* genome (Hori et al., 2014). In contrast to xylans, MLG is not a common cell wall component of flowering plants and is restricted to Poales, horsetails, some liverworts, red and brown algae and the CGA (Eder et al., 2008; Fry et al., 2008b; Sørensen et al., 2008; Salmeán et al., 2017; Popper and Tuohy, 2010). In young *Zygnema* S, a low concentration of MLG was present in outer cell walls, while the signal increased in old filaments and was particularly strong in walls of individual cells. A similar trend was observed in *Klebsormidium* (Fig. S2), where the epitopes were restricted to only a few cells. Occasionally, binding of mAb 400-3 was detected close to cross cell walls. Correspondingly, binding of mAb 400-3 in *Klebsormidium* was mostly restricted to the cell corners between individual cells. As shown for *Equisetum*, the MLG content correlates positively with developmental stage, since it predominates in secondary cell walls (Leroux et al., 2011; Sørensen et al., 2008). The restriction of MLG to secondary cell walls was also found in the green alga *Micrasterias* (Zygnematophyceae; Eder et al., 2008). Thus, incorporation of MLG in old filaments of *Klebsormidium* and *Zygnema* S might be an age-dependent process.

### Different transglycosylase activities are associated with culture age

Extracts prepared from young (1 month) and old (1 year) *Klebsormidium* and *Zygnema* S cultures and field-collected *Chara* sp. were capable of incorporating XyGO-SR, therefore they exhibited xyloglucan endotransglycosylase activity. When compared with *Klebsormidium* and *Chara*, *Zygnema* S extracts showed the most lax donor substrate specificity (Fig. 2, Table 1). The restriction of transglycosylase activity toward MLG (i.e. MLG:xyloglucan endotransglucosylase activity; MXE) in old *Zygnema* S extracts is interesting because it corresponds to findings in *Equisetum* sp., where older tissues exhibit higher MXE:XET rates than younger tissues (e.g. young compared to old stems; Fry et al., 2008b; Mohler et al., 2013). This might be related to the higher MLG content in secondary cell walls of old *Equisetum* tissues (see previous paragraph). This suggests that MLG, and its processing by MXE action, may perform similar functions in *Equisetum* and Zygnematophyceae but not Klebsormidiophyceae (i.e. cell wall strengthening of older cells/tissues) (Fry et al., 2008b).

Intriguingly our cultures exhibited two further novel transglycosylase activities: (1) between xyloglucan and mannans (*Klebsormidium*, *Zygnema* S), and (2) between xyloglucan and xylan (*Zygnema* S). The capability to act on galactomannan is particularly interesting because, although mannan transglycosylase activity, which crafts mannan-based plant polysaccharides including galactomannan onto galactoglucomannan oligosaccharides, has been found in various land plants (Schröder et al., 2004), to our knowledge, no previous studies have reported transglycosylase reactions between galactomannan (donor) and xyloglucan oligosaccharides (acceptor) (McGregor et al., 2017). Although this novel activity needs further investigation, the ability of algae to process mannans by transglycosylation is plausible. By using immunolabelling techniques, we found mannans to occur abundantly in both *Klebsormidium* and *Zygnema* S cell walls. This confirms previous studies where mannans were detected in the cell walls of CGA in glycan microarray experiments (Sørensen et al., 2011). Additionally, although members of the CGA were not investigated, a recent study highlights the ancient evolution of the Endo Glucanase 16 (EG16) clade [within Glycoside Hydrolase family 16 (GH16)] as a class of enzymes that are capable of carrying out heterotransglycosylation reactions and have a broad substrate specificity (McGregor et al., 2017).

### XET localisation is related to morphology

Localisation studies on *Klebsormidium* and *Zygnema* S filaments taken from young and old cultures suggest that transglycanase action is involved into the construction and growth of longitudinal cell walls. The main donor substrates might be xyloglucan and/or mannans, because epitopes of these hemicelluloses colocalised predominately with the sites of enzyme action. The idea that transglycanase activity is involved in cell wall growth of the species investigated is supported by the following observations: (1) that enzyme action is not detectable in cross cell walls, (2) that it is abundant in longitudinal cell walls of young filaments exhibiting expanding cells, but (3) that it is absent in thick-walled cells (pre-akinetes) of old *Zygnema* S filaments that had ceased growth. However, (4) transglycanase action was present in smaller (i.e. expanding) cells within the same filaments. In contrast to *Klebsormidium* and *Zygnema* S, transglycanase action in *Chara* was also found between individual cells (i.e. all cell planes). A tempting speculation is that these differences are linked to different body plans (parenchymatous versus filamentous) and mechanisms of cell division (phragmoplast versus cleavage/reduced phragmoplast; Staehelin and Hepler, 1996). *Chara* and land plants use a ‘true phragmoplast’, while cell division in *Zygnema* S and *Klebsormidium* occurs perpendicular to the length axis of the uniserate filaments by forming a centripetally encroaching septum between two daughter cells (Graham et al., 2000). Whereas in Klebsormidiophyceae centrosomes organize mitotic spindles, in Zygnematales, centripetal furrowing can be accompanied by the formation of a small cell plate in the cell centre, which is connected to the appearance of a rudimentary phragmoplast (Sawitzky and Grolig, 1995; Scherp et al., 2001; Yoon et al., 2010). Since Zygnematophyceae are considered the sister group to land plants, it has been proposed that the different mechanisms of cell division between Charophyceae/land plants and Zygnematophyceae result partially from reductions (e.g. less complex phragmoplast) in the latter (Buschmann and Zachgo, 2016). One such modification might be that XET is not involved in the construction of the cell plate as found in land plants, where xyloglucan occurs in the equatorial plane during late anaphase and shows a strong spatial correlation with XET action during cell plate formation (Yokoyama and Nishitani, 2001). Interestingly, similar to what is seen for cell plates, *Chara* cell walls are rich in non-methylesterified homogalacturonan (HG) and contain only low amounts of xyloglucan and cellulose (Sørensen et al., 2011). As proposed by Proseus and Boyer (2008), the ‘pectate cycle’ mediates non-enzymatic anisotropic growth of *Chara* cells, involving incorporation of HG and the formation of new Ca^2+^–HG links. Young *Chara* cells predominantly incorporate xyloglucan oligosaccharides into their cell walls, suggesting that transglycanase action is nevertheless involved in cell growth as well and accompanies the ‘pectate cycle’.

### Hydrolytic activities also change with cell age

As well as transglycosylases, plants contain numerous enzymes that use water as an acceptor substrate resulting in the hydrolysis of a polysaccharide. Franková and Fry (2011) screened enzyme extracts from more than 50 land plants and revealed a variety of hydrolytic activities, including β-d-xylosidase, endo-(1→4)-β-d-xylanase, β-d-mannosidase and endo-(1→4)-β-d-mannanase, α-d-xylosidase activities. As shown by a viscometric assay, extracts prepared from young cultures caused more rapid scission of xyloglucan, MLG and xylan compared with extracts from older cultures. However, when galactomannan was added as a substrate, old *Klebsormidium* and *Zygnema* S extracts had higher hydrolytic activities. Mannose-containing polysaccharides are considered among the main hemicelluloses in CGA (Popper, 2008). Land plant mannans serve numerous biological functions (Liepmann et al., 2007 and references therein), including as structural elements and energy reserves (Moreira and Filho, 2008). Thus, it is possible that the high capability of both *Klebsormidium* and *Zygnema* S to degrade mannans allows mobilization of energy reserves. Old filaments of both *Klebsormidium* (K.H., unpublished data) and *Zygnema* S (Herburger et al., 2015), have considerably lower photosynthetic performance compared with young filaments, as shown by microscopic Imaging-PAM (Fig. S4). Thus, mannans in old filaments might serve as an additional easily accessible energy reservoir, and cover along with lipids (Pichrtová et al., 2016b) the high metabolic costs when pre-akinetes start germinating, accompanied by a high cell division rate (Pichrtová et al., 2016a). Furthermore, mannans (and/or other cell wall polysaccharides) might be partially removed from the cell wall by hydrolysis to gain the building blocks for newly formed cell wall areas.

### Functional role of hemicelluloses in filamentous CGA

The specific occurrence of some hemicelluloses in the contact zone of individual cells (MLG, xylans) and cross cell walls (xyloglucan, mannans) of *Zygnema* S and *Klebsormidium* supports the hypothesis that these polysaccharides play an important role in cell–cell attachment (Ikegaya et al., 2008). Treating *Spirogyra* sp. (Zygnematophyceae) filaments with cellulase or removing pectin from the cell wall does not cause cell detachment; however, adding exogenous xyloglucan promotes attachment of the cell wall to experimentally induced rhizoids, suggesting that xyloglucan might be involved in cell–cell attachment (Ikegaya et al., 2008). Furthermore, cell detachment did not increase following PL treatment of either *Klebsormidium* or *Zygnema* S. Thus, pectins (homogalacturonan) might be important for the attachment of algae to surfaces (Domozych et al., 2014), and certainly for the mucilage production in Zygnematophyceae (e.g. Eder et al., 2008; Eder and Lütz-Meindl, 2010), but not for cell–cell attachment. PL treatment did not influence binding of the mAbs LM15 and 400-4 to the cell walls of *Klebsormidium*. This is perhaps not surprising since, although genes involved in the homogalacturonan biosynthesis occur in *Klebsormidium flaccidum* (Hori et al., 2014), they may not be (highly) transcribed and the pectin fraction of *Klebsormidium* lacks high amounts of galacturonic acid (Domozych et al., 1980; O'Rourke et al., 2015) and homogalacturonan epitopes (Sørensen et al., 2011).

### Conclusion

The present study reports the first *in vivo* determination of the sites of transglycanase action for xyloglucan as acceptor substrate in charophyte green algae (CGA). Additional (hetero)-transglycanase activities were found to exist in CGA members between xyloglucan and (1) MLGs, (2) xylans or (3) mannans. Although CGA have similar cell wall compositions to those of land plants, they exhibit conspicuous structural and chemical changes, including in transglycanase specificities, in response to ageing and stress. Long-term cultivation experiments allowed us to gain new insights into algal cell wall metabolism showing that the hemicellulose content and distribution change, and that transglycanase action is more abundant in young filaments of *Klebsormidium* and *Zygnema* S. Furthermore, transglycanase action appeared to be associated with morphology as it was restricted to longitudinal cell walls, where cell expansion occurs in filamentous CGA (*Klebsormidium* and *Zygnema* S), but was found in anticlinal and periclinal cell walls in parenchymatous *Chara*.

## MATERIALS AND METHODS

### Algal material and long-term cultivation

Young (1 month) and old (1 year) cultures of *Zygnema* sp. ‘Saalach’ (‘*Zygnema* S’; SAG 2419; Herburger et al., 2015) and *Klebsormidium crenulatum* (‘*Klebsormidium*’; SAG 2415; Karsten et al., 2010) were maintained on 1.5% agar plates or in 250 ml Erlenmeyer flasks (subsamples of old *Klebsormidium*). *Zygnema* S was cultivated in Bold's Basal Medium (BBM, Bischoff and Bold, 1963) and *Klebsormidium* in modified BBM (3 NMBBM; Starr and Zeikus, 1993). Culture conditions were described in detail elsewhere (Herburger et al., 2015). *Chara* sp. were collected from Eglington canal, Galway (53°16′35.1″N 9°03′32.1″W), in September 2015 and January 2016 and washed in BBM to remove any co-occurring algae and bacteria. The *Chara* specimens were then viewed under a light microscope, and undamaged and uncontaminated specimens selected for analysis.

### Preparation of alcohol-insoluble residue and cell wall fractioning

The alcohol-insoluble residue (AIR) was prepared according to O'Rourke et al. (2015). Filaments of young and old *Zygnema* S and *Klebsormidium* (0.8–1 g fresh mass) were washed thoroughly with distilled water (dH_2_O), frozen in liquid nitrogen, ground with a mortar and pestle, stirred in five volumes of 70% ethanol containing 1% (v/w) formic acid for 16 h and centrifuged at 5000 ***g*** for 10 min. The pellet was washed five times in 70% ethanol, once in acetone and was then air dried. The AIR was stirred in phenol:acetic acid:water (2:1:1, w/v/v) at 70°C for 1 h, washed in ethanol to remove proteins and separated into six fractions according to O'Rourke et al. (2015) [i.e. two pectin fractions (extracted in ammonium oxalate at 100°C for 2 h or 16 h), hemicellulose A (insoluble in 6 M NaOH after 72 h) and B (soluble in NaOH), pooled washings (‘wash’; soluble in buffer, pH 4) and the inextractable residue (‘α-cellulose’)].

### Enzyme extraction

Total buffer-extractable protein from young and old *Zygnema* S and *Klebsormidium* was prepared according to Fry et al. (2008a). Briefly, 0.9–1.5 g of algal fresh mass was ground in 4.8–8 ml ice-cold extraction buffer [10 mM CaCl_2_, 300 mM Na succinate (pH 5.5), 2 mM ascorbate, 15% (v/v) glycerol, 3% (w/v) polyvinylpolypyrrolidone], kept on ice for 2 h, filtered through Miracloth (Merck Millipore, Tullagreen, Carrigtwohill, Ireland) and centrifuged at 12,000 ***g*** for 10 min (4°C). Extracts were dialysed against dH_2_O and either used immediately for *in vitro* assays or stored at −20°C.

### Source of polysaccharides for dot-blot and viscometric assays

Tamarind xyloglucan, carob galactomannan (high viscosity), beechwood xylan, mixed-linkage (1→3,1→4)-β-glucan (MLG) from oat (high viscosity), barley (high viscosity) and Icelandic moss (‘lichenan’) were purchased from Megazyme (Wicklow, Ireland), and xanthan gum and locust bean gum from *Ceratonia siliqua* seeds from Sigma-Aldrich (Steinheim, Germany). Arabinogalactan proteins (AGPs) were extracted from young carrots as described by Popper (2011).

### Transglycosylase activity – dot-blot assay

A fluorescent dot-blot assay was used to estimate transglycosylase activity *in vitro* (Fry, 1997; Chormova et al., 2015). Whatman No. 1 filters (Whatman, Dassel, Germany) were coated with nine different biologically relevant cell wall polysaccharides [1% (w/v) in dH_2_O; see previous paragraph], left to dry and coated with sulphorhodamine-labelled xyloglucan oligosaccharides (XyGO-SRs; ∼5 µM) prepared according to Kosík and Farkaš (2008) from enzymatically digested tamarind xyloglucan (Sulová et al., 1995). Test papers were loaded with 5 µl of algal enzyme extract and incubated in darkness at ∼20°C between acetate sheets to maintain humidity for 2 h. Papers were washed in ethanol:formic acid:water (1:1:1, v/v/v) for 1.5 h, rinsed twice with dH_2_O and dried overnight. Orange fluorescence emitted by bound XyGO-SR was visualized by using a CX-20 work station [excitation 365 nm; Spectronics Corp., Westbury (NY), USA] connected to a Nikon Coolpix 8400 camera (Nikon Corp., Tokyo, Japan). Test papers lacking XyGO-SR or polysaccharide coating, or loaded with heat-inactivated enzyme extracts served as controls.

### *In vivo* localization of transglycosylase activity

*In vivo* incorporation of XyGO-SR into algal cell walls was visualized according to Vissenberg et al. (2000) with modifications. Young and old filaments of *Zygnema* S or *Klebsormidium* or freshly collected *Chara* sp. were incubated in 1 ml culture medium (*Zygnema* S and *Klebsormidium*, BBM or 3 NMBBM, pH 5.5; *Chara* media described by Zhu and Boyer, 1992) containing 5 µM XyGO-SR for 2 h. Filaments were washed with ethanol:formic acid:water (6:0.4:4, v/v/v) for 10 min and with 5% (v/v) formic acid overnight. *Zygnema* S and *Klebsormidium* filaments were rinsed twice with culture medium and incubated in 1 ml dimethylformamide (DMF) either for 2 min (*Klebsormidium*) or 4 min (*Zygnema* S) to reduce autofluorescence by extracting photosynthetic pigments (Fig. S1). *Chara* were washed twice with culture medium. Incorporated XyGO-SR in *Zygnema* S and *Klebsormidium* was visualized with a Zeiss Pascal 5 confocal laser-scanning microscope (CLSM) equipped with an argon laser [excitation 488 nm, emission 560 nm long pass (LP), false colour green] on a Zeiss Axiovert 200 M microscope. A corresponding bright-field image was collected in a second channel. Incorporated XyGO-SR in *Chara* sp. was visualised by using the DAPI channel (excitation, 320–390 nm; emission, 430–490 nm) of an Olympus 1X51, X-Cite series 120 and imaged using an Olympus DP71 camera. Control groups contained xyloglucan oligosaccharides lacking the sulphorhodamine group.

### Immunolabelling

Young and old filaments of *Zygnema* S and *Klebsormidium* were chemically fixed [3% (v/v) paraformaldehyde, 1 h], blocked [with 1% (w/v) bovine serum albumin (BSA) for 1 h; Sigma-Aldrich], washed and incubated under continuous shaking for 2 h in monoclonal antibodies [mAbs; 1:6 in phosphate buffered saline (PBS)] purchased from Biosupplies (400 series) or Plant Probes (LM series). The mABs bind to xyloglucan (LM15; Marcus et al., 2008), (1→4)-β-mannan (400-4; Pettolino et al., 2001), mixed-linkage (1→3, 1→4)-β-d-glucan (400-3; Meikle et al., 1994) or to unsubstituted/low-substituted (1→4)-β-xylan (LM10; McCartney et al., 2005). Filaments were blocked again [0.5% (w/v) BSA, 30 min] and incubated in the secondary antibody (1:100 in PBS for 2 h): Alexa Fluor 488-conjugated goat anti-mouse IgG (g1) (Thermo Fisher Sci., Waltham, MA) for 400-3 and 400-4; FITC goat anti-rat-IgG (whole molecule) (Sigma-Aldrich) for LM10 and LM15. To test whether epitopes are masked by pectins preventing binding of mAbs (Marcus et al., 2008) enzymatic unmasking by incubating algal filaments in 4 units ml^−1^ pectate lyase for 18 h (PL; E-PLYCJ, Megazyme) prior to chemical fixation was performed (Domozych et al., 2014). Filaments were examined with a confocal laser-scanning microscope (excitation, 488 nm; emission, 505–550 nm band pass, false coloured green and 560 nm long pass, false coloured red). Up to 50 optical sections through a filament allowed generation of *z*-stacks. A corresponding bright-field image was collected in a third channel and merged with the false colour red image. As a control, the primary antibody was omitted or heat-inactivated prior use.

### Cryofixation and labelling of semi-thin sections

Cryofixation using a Leica EMPACT high-pressure freezer (Leica Microsysteme), freeze substitution in a Leica EM AFS and embedding in LR-White (London Resin Company Ltd.) was carried out according to Lütz-Meindl and Aichinger (2004). From fixed material (1-month-old filaments of *Zygnema* S or *Klebsormidium*), semi-thin sections were prepared by using a Leica Ultramicrotome (Leica Microsystems GmbH), transferred to ten-well polylysine-coated slides (Thermo Fisher Scientific), and labelled with the mAbs LM15 and 400-4 as described in Herburger and Holzinger (2015). Some sections were enzymatically unmasked by PL (E-PLYCJ) incubation. Fluorescence of secondary antibodies (see previous paragraph) was visualized with a confocal laser-scanning microscope.

### Viscometric assay of hydrolytic activity of enzyme extract

Hydrolytic activity of algal enzyme extracts was tested with a viscometric assay as described by Fry (1998). Reaction mixtures contained 10% (v/v) enzyme extract and 1% (w/v) polysaccharide (xyloglucan, MLG, galactomannan or xylan) in buffer (10 mM CaCl_2_, 300 mM Na succinate, pH 5.5). Mixtures were sucked into a 1 ml vertical glass pipette and an efflux time of 0.8 ml liquid as a function of incubation time up to 1 day was monitored. Efflux time was expressed as percentage of control assays, which contained heat-inactivated enzyme extracts.

### Microscopic Imaging-PAM

The effective quantum yield of PSII [Y(II), 620 nm] and near-infrared remission (NIR, 780 nm) of young (1 month culture) and old (1 year culture) *Zygnema* S and *Klebsormidium* filaments were visualised with an Imaging-PAM (M-series, Heinz Walz GmbH) connected to a modified Axio Scope A.1 epifluorescence microscope equipped with a Zeiss Fluar 40×1.3 NA objective and CCD Camera IMAG-K6 (Herburger and Holzinger, 2015).

## Supplementary Material

Supplementary information
