# Supplementary material for: Localisation and substrate specificities of transglycanases in charophyte algae relate to development and morphology
Source: J Cell Sci. 2018 Jan 15;131(2):jcs203208. doi: 10.1242/jcs.203208 (PMC5722204; doi:10.1242/jcs.203208)
Supplement: Supplementary information [file joces-131-203208-s1.pdf]

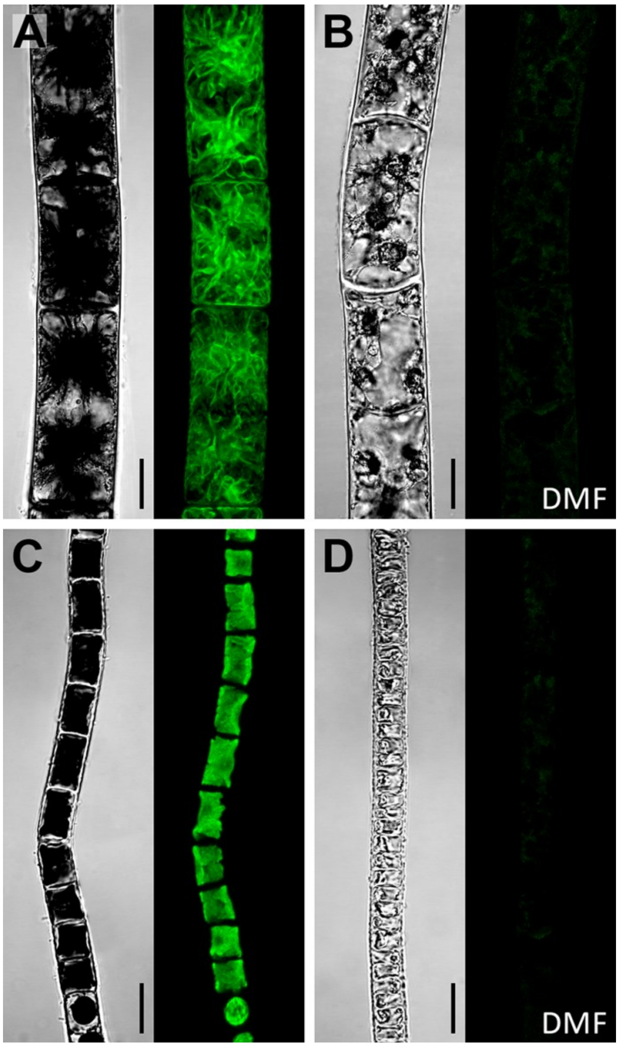

Fig. S1. Effect of dimethylformamide (DMF) on chloroplast auto-fluorescence. CLSM micrographs of *Zygnema* S (A, B) and *Klebsormidium crenulatum* (C, D) filaments before (A, C) and after (B, D) incubation in DMF. Bars = 10 µm.

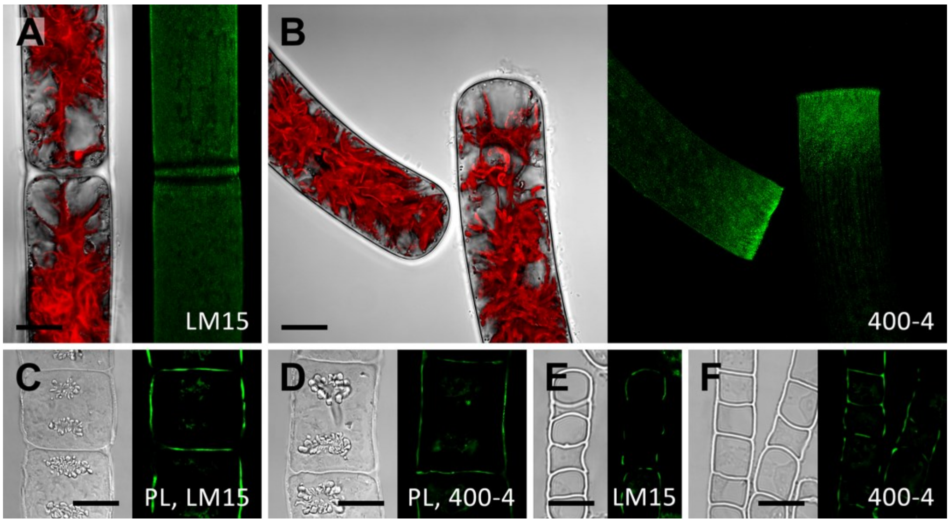

Fig. S2. Immunolabelling of whole cells and semithin sections (CLSM micrographs). *Zygnema* S (A-D) and *Klebsormidium crenulatum* (E, F) filaments labelled with the monoclonal antibodies LM15 or 400-4. Whole cell labelling (A, B), labelling of semithin sections (C-F). Red autofluorescence is shown. (A) Weak fluorescence in outer and cross cell walls. (B) Recently fragmented filament with fluorescence close to terminal cross cell walls. (C-F) Labelling in outer and cross cell walls. Bars 10 = µm

Journal of Cell Science • Supplementary information

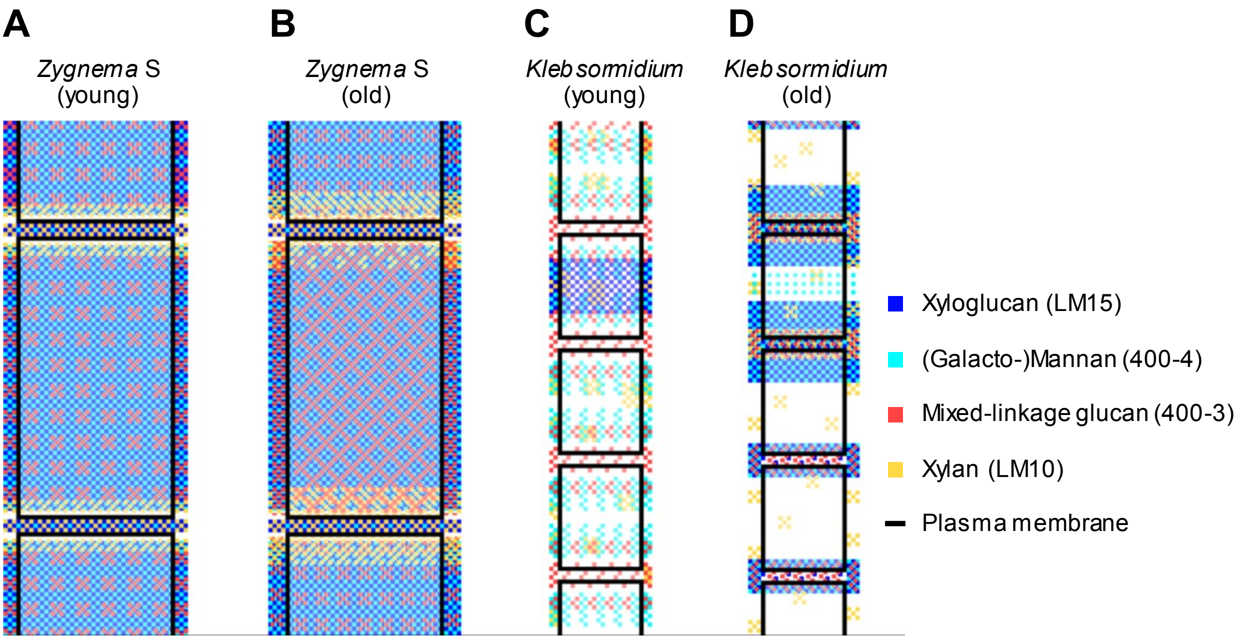

Fig. S3. Hemicellulose distribution in filamentous charophytes. Schemes are based on immuno-localisations of hemicellulose epitopes (antibody names in brackets) in young (1 month) and old (12 months) filaments of (A, B) *Zygnema* S and (C, D) *Klebsormidium crenulatum*.

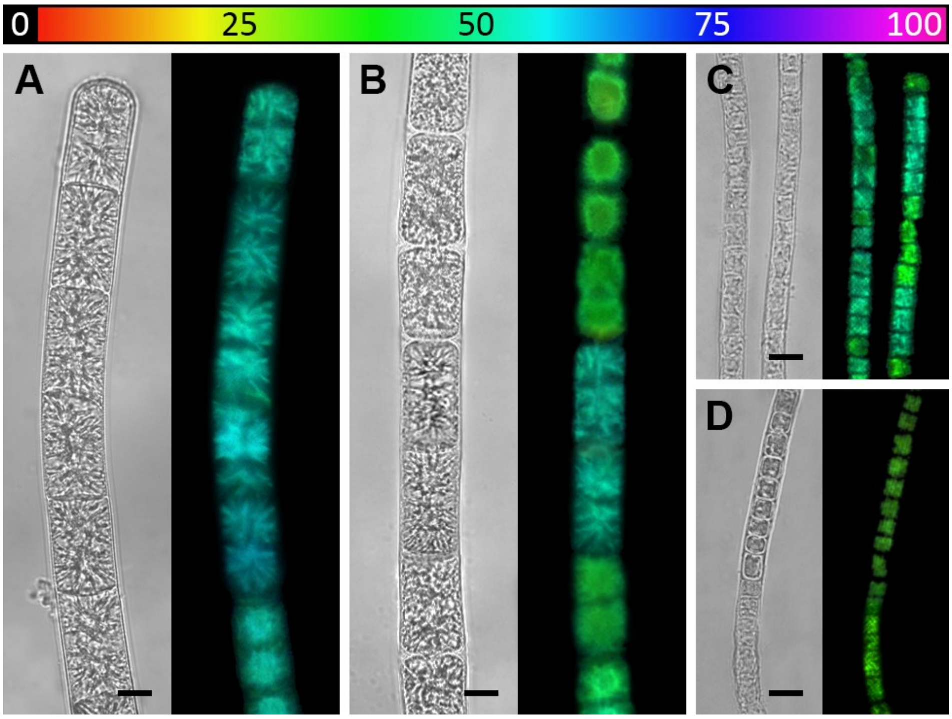

Fig. S4. Photosynthetic performance of *Zygnema* S and *Klebsormidium crenulatum*. NIR and corresponding Y(II) images (false colour) of young (A, C) and old (C, D) filaments of *Zygnema* S (A, B) and *Klebsormidium* (C, D). The colour bar at the top indicates the relative Y(II) as a percentage. (A) Vacuolated cells containing two stellate chloroplasts and high Y(II) values (~0.64). (B) Akinete cells dominate old filaments and exhibit a lower Y(II) (i.e. ~0.41) than younger (i.e. vacuolated, thin-walled, growing) cells within the same filament (~0.6). (C) Cells of young filaments with a higher Y(II) (~0.48-0.59) than cells of old filaments (D) (~0.3). Bars = 10 µm.

Tab. S1 Evidence for major cell wall polysaccharides in Charophytes.

Cell wall component	Klebsormidiophyceae	Charaphyceae	Zygnematophyceae
Cellulose	A[1,5]B[7]C[6]	A[5]B[8,7]	A[5]B[3,5,7]
Callose	A[5]B[3,5]C[6]	A[5]B[5,8]	A[5]B[3]
**Pectins**			
HG	A[1,5*]B[5]C[6]	A[5,7]B[8]	A[5,7]B[4,9,10]
RGI/RGII	-	A[5,7†]	A[7†]
**Hemicelluloses**			
Xyloglucan	B[5]C[6]	A[5]B[5,8]	B[5,8]
MLG	B[5]	B[5,8]	A[4,5]B[4,5]
Mannans	B[5]	B[5,8]	B[5]
Xylans	B[5]	B[5,8]	B[5]
**(Glyco)Proteins**			
Arabinogalactan proteins	-	B[5]	B[5]
Expansins	C[6]	C[11]	C[11]
Extensins	C[12]	-	B[5]

Key: A biochemical, B immunological/histochemical, C genetically (genomic/transcriptomic data), '-' no evidence.

*Biochemical evidence for HG in Klebsormidiophyceae is ambiguous. While Domozych et al. (1980) and Sorensen et al. (2011) detected galacturonic acid in *K. flaccidum*, O'Rourke et al. 2015 suggested its absence in *K. flaccidum* and *K. subtile*.

[†]Suggested by the co-occurrence of 1→5-Ara *f*, 1→4-Gal *p* and 2→4-Rha *p*.

[1]Domozych et al. 1980, [2]Popper and Fry 2003, [3]Herburger and Holzinger 2015, [4]Eder et al. 2008, [5]Sørensen et al. 2011, [6]Hori et al. 2011, [7]O'Rourke et al. 2015, [8]Domozych et al. 2009, [9]Domozych et al. 2006, [10]Eder and Lütz-Meindl 2010, [11]Vannerum et al. 2011, [12]Liu et al. 2016

Referneces not in the main document:

- Domozych, D. S., Sørensen, I. and Willats, W. G. (2009). The distribution of cell wall polymers during antheridium development and spermatogenesis in the Charophycean green alga, Chara corallina. Ann. Bot. 104, 1045-1056.
- Liu, X., Wolfe, R., Welch, L. R., Domozych, D. S., Popper, Z. A. and Showalter, A. M. (2016). Bioinformatic identification and analysis of extensins in the plant kingdom. PLoS ONE 11, e0150177.
- Popper, Z. A. and Fry, S. C. (2003). Primary cell wall composition of bryophytes and charophytes. Ann. Bot. 91, 1-12.
- Vannerum, K., Huysman, M. J., De Rycke, R., Vuylsteke, M., Leliaert, F., Pollier, J., Lü tz-Meindl, U., Gillard, J., De Veylder, L., Goossens, A. et al. (2011). Transcriptional analysis of cell growth and morphogenesis in the unicellular green alga Micrasterias (Streptophyta), with emphasis on the role of expansin. BMC Plant Biol. 11, 128.
